# Repurposing Therapeutic Drugs Complexed to Vanadium in Cancer

**DOI:** 10.3390/ph17010012

**Published:** 2023-12-21

**Authors:** Ana Luísa De Sousa-Coelho, Gil Fraqueza, Manuel Aureliano

**Affiliations:** 1 Algarve Biomedical Center Research Institute (ABC-RI), Universidade do Algarve, 8005-139 Faro, Portugal; 2 Algarve Biomedical Center (ABC), Universidade do Algarve, 8005-139 Faro, Portugal; 3 Escola Superior de Saúde, Universidade do Algarve (ESSUAlg), 8005-139 Faro, Portugal; 4 Instituto Superior de Engenharia (ISE), Universidade do Algarve, 8005-139 Faro, Portugal; gfraque@ualg.pt; 5 Centro de Ciências do Mar (CCMar), Universidade do Algarve, 8005-139 Faro, Portugal; 6 Faculdade de Ciências e Tecnologia (FCT), Universidade do Algarve, 8005-139 Faro, Portugal

**Keywords:** drug repurposing, vanadium complexes, vanadate, decavanadate, cancer treatment

## Abstract

Repurposing drugs by uncovering new indications for approved drugs accelerates the process of establishing new treatments and reduces the high costs of drug discovery and development. Metal complexes with clinically approved drugs allow further opportunities in cancer therapy—many vanadium compounds have previously shown antitumor effects, which makes vanadium a suitable metal to complex with therapeutic drugs, potentially improving their efficacy in cancer treatment. In this review, covering the last 25 years of research in the field, we identified non-oncology-approved drugs suitable as ligands to obtain different vanadium complexes. Metformin-decavanadate, vanadium-bisphosphonates, vanadyl(IV) complexes with non-steroidal anti-inflammatory drugs, and cetirizine and imidazole-based oxidovanadium(IV) complexes, each has a parent drug known to have different medicinal properties and therapeutic indications, and all showed potential as novel anticancer treatments. Nevertheless, the precise mechanisms of action for these vanadium compounds against cancer are still not fully understood.

## 1. Introduction

Several metals and metal complexes such as platinum, gold, and ruthenium, and complexes containing essential metals such as Mn, Cu, and Co, show potential for application in medicine [1,2,3]. Vanadium, although yet to be fully investigated, displays specific versatile properties that allow it to form several types of complexes and compounds with distinct biological applications. These have been investigated as potential therapeutic agents against relevant first-world diseases such as diabetes, cancer, and neurodegenerative and other aging-related diseases [4,5].

Although there has been an exponential increase in the number of papers published in the field over the last few decades, further studies are still required to fully comprehend the mechanisms of action of metal-based drugs. For vanadium compounds, several mechanisms of action have been proposed [6,7], namely the effects of vanadium on oxidative stress and lipid peroxidation [8]. In addition to being a transition metal, and thus inducing Fenton-like reactions, vanadium has the ability to form polyoxidovanadates (POVs) that can target several biomolecules and affect essential biochemical processes. Some of these processes have direct and/or indirect associations with oxidative stress, aging, and diseases, although the major mechanisms of action of vanadium and metals in general are yet to be completely understood.

Although past research has studied the application of vanadium-based compounds in clinical practice, there is a need to further explore their potential against cancer diseases. The repurposing of clinically approved drugs, complexed as metal-based drugs, may represent a simple approach to close the gap and increase the number of vanadium metallodrugs for cancer treatments. Hence, the goal of this review was to cover the last 25 years of research to identify approved drugs usable as ligands in different vanadium complexes and describe their proposed mechanisms of action in treating cancer, highlighting novel potential therapeutic candidates based on drug repurposing.

## 2. Drug Repurposing in Cancer

The number of articles published related to drug repurposing increased substantially over the last decade [9]. Drug repurposing, or repositioning, refers to the identification and application of clinically approved drugs to alternative disease indications and new therapeutic purposes. Because of their established formulations, known adverse effect profiles, and defined pharmacokinetic properties, the re-use of such existing therapies for new indications can save the time and money otherwise invested for the de novo drug design and development [10]. Repurposed therapies for cancer patients allow for faster treatment with fewer restrictions due to safety concerns. Additionally, drugs that do not display direct cytotoxicity may be combined to target distinct critical pathways, potentially producing a synergistic therapeutic effect or allowing tailored regimens, increasing the number of patients who will benefit from precision medicine [11]. The re-use of existing oncological drugs for new oncological indications (i.e., different types of cancers), is referred to as “soft repurposing”, whereas the use of non-cancer drugs as anticancer medications is referred to as “hard repurposing” [12].

Drugs from many different pharmacotherapeutic classes may be amenable to repositioning. For instance, antiparasitic (ex.: mebendazole) and antiepileptic (ex.: valproate) drugs are potentially novel options for glioblastoma based on their ability to cross the blood–brain barrier [13,14,15]. However, there is greater evidence for the repurposing of antidiabetic and antihypertensive drugs [16,17], given their efficacy in chronic metabolic diseases (diabetes and cardiovascular disease), because (1) these diseases are highly prevalent and their treatments well-studied, with an increased amount of knowledge regarding their use and potential benefit; (2) there are metabolic links and shared risk factors between these diseases and cancer; and (3) drugs that can be administered chronically are normally well-tolerated [18].

Repurposed drugs are emerging as promising strategies to overcome therapy resistance, one of the greatest challenges of current cancer treatments. If the molecular pathways driving drug resistance are identified, drugs that specifically target those pathways will be a great asset. For instance, it is expected that when combined with immunotherapies, repurposed drugs that modulate the immunosuppressive tumor microenvironment (TME), will boost their effect and avoid therapeutic failure [19,20,21]. This means that optimized approaches are needed to identify “old” candidate drugs with such actions. Indeed, the range of computational predictive tools, high-throughput screening methods, machine learning algorithms, bioinformatics analysis, and artificial intelligence that facilitate the drug repurposing process, unraveling molecular signatures, and contribute to novel, affordable, and tailored treatment options, is very impressive [22,23,24,25].

## 3. The Potential of Vanadium for Cancer Therapeutics

Many vanadium complexes show therapeutic potential in cancer. Involved pathways for vanadium compounds may include AMP-activated protein kinase (AMPK) activation and protein tyrosine phosphatase 1B (PTP1B) inhibition pathways, as described for the treatment of breast cancer [26]. Polyoxidovanadates (POVs) inhibit the activity of P-type ATPases [27,28,29] and oxygen consumption in the mitochondria [30]. Meanwhile, changes in lipid peroxidation may be one of the mechanisms involved in the anticancer action of vanadium [8]. In melanoma, several vanadium compounds and/or materials lead to decreased cell viability, changes in cell morphology and apoptosis, cell cycle arrest, production of reactive oxygen species (ROS), inhibition of mitochondrial respiration, differential expression of proteins and signaling, and tumor regression along with increased survival rates in animal models [31]. Over the last few years, significant evidence has been gathered supporting the application of different vanadium complexes as anticancer agents, which is extensively reviewed elsewhere [32,33,34,35,36].

## 4. Vanadium Complexed with Marketed-Approved Drugs

**Figure 1:**
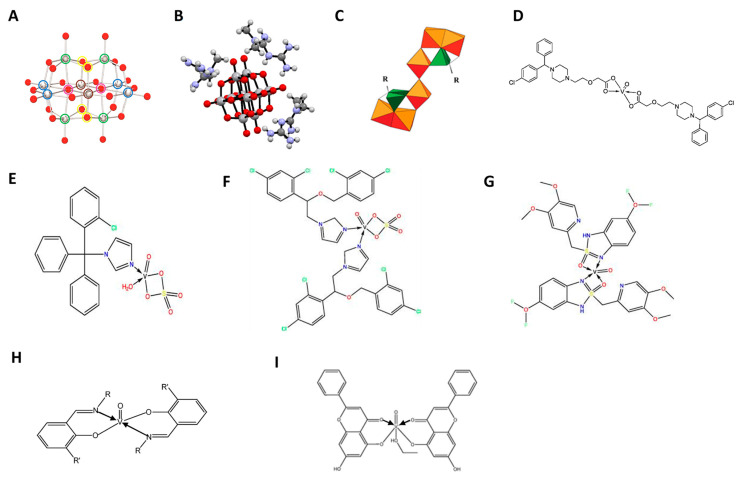
Structures of vanadium compounds and complexes with approved drugs. (**A**) Structure of the decameric species of vanadate, decavanadate, V_10_O_28_^6-^. Color code: V, gray; O, red. The green (four), blue (four), and brown (two) circles refer to vanadium atoms with the same chemical environment [41]; (**B**) Ball and stick representation of metforminium decavanadate (H_2_Metf)_3_[V_10_O_28_]·8H_2_O. Water molecules are omitted for clarity [42]; (**C**) Polyhedral representation common to the Mo_6_L_2_ (where L corresponds to a ligand, either alendronate (Ale) or zoledronate (Zol)) POM frameworks, green tetrahedral = PO_3_C, orange polyhedra = MoO_6_ [43]; (**D**) Oxidovanadium(IV) complexes with cetirizine, [VO(CTZ)_2_] 2H_2_O [44]; (**E**) Clotrimazole oxidovanadium(IV) complex [VO(SO_4_)(CTNZ)(H_2_O)]H_2_O; (**F**) Miconazole oxidovanadium(IV) complex, [VO(SO_4_)(MNZ)_2_] H_2_O; (**G**) Pantoprazole oxidovanadium(IV) complex, [VO(PNZ)_2_]SO_4_.2H_2_O; (**H**) Oxidovanadium(IV) complexes with Schift based compounds, such as for ibuprofen and naproxen [45]; (**I**) Oxidovanadium(IV) chrysin complex [46].

Vanadium-based complexes may incorporate different ligands, ranging from chemical elements such as cobalt to plant pigments (flavonoids) [37,38], or synthetic drugs, enclosing defined pharmacological properties and indications [39]. Complexes of metal ions with free drugs may reduce the toxicity of the drugs and increase their lipophilicity, improving their transport across cell membranes [40]. In this review, the effects of several vanadium compounds and complexes with distinct chemical structures are described (Figure 1).

### 4.1. Vanadyl(IV) Complexes with Non-Steroidal Anti-Inflammatory Drugs

Tumor-promoting inflammation is one of the hallmarks of cancers, along with many others, such as avoiding immune destruction [47]. The connection of inflammation with tumor development and progression may justify the interest in exploring anti-inflammatory drugs in cancer research. Studies regarding their repurposing have shown their potential as chemopreventive agents against certain types of cancer or as anticancer agents [48]. In fact, non-steroidal anti-inflammatory drugs (NSAIDs) may protect against the development of cancer, as studied for aspirin and ibuprofen at low doses [49].

**Figure 2:**
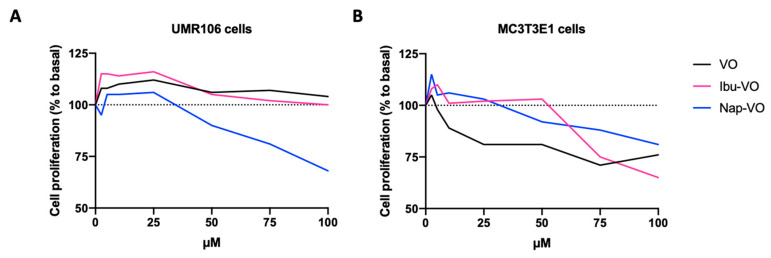
Effects of Ibu-VO, Nap-VO, and VO, on UMR106 (**A**) and MC3T3E1 (**B**) cell proliferation. Approximate values were extracted from [51] and are expressed as a percentage of the basal value (without treatment, 0 µM). Abbreviations: Ibu-VO, vanadyl(IV) complexes with ibuprofen; Nap-VO, vanadyl(IV) complexes with naproxen; VO, vanadyl(IV).

Notably, such NSAIDs have a carboxylate group available for metal–ligand interaction, which has raised interest in their use in complexes with vanadium for medicinal applications [50]. Different vanadyl(IV) complexes with either ibuprofen (2-(4-isobutylphenyl)propionic acid) ([VO(Ibu)_2_].5CH_3_OH) or naproxen (6-methoxy-α-methyl-2-naphthalene acetic acid) ([VO(Nap)_2_].5CH_3_OH) have been synthesized [51]. All NSAIDs–VO^2+^ complexes (Figure 1H) were then characterized with respect to their potential effect on the proliferation of osteoblast-like cells [51]. The results of the mitogenic bioassay with increasing concentrations of NSAIDs-VO^2+^, in both tumoral UMR106 from a rat osteosarcoma (Figure 2A) and non-transformed MC3T3E1 derived from mouse calvaria (Figure 2B), showed in some cases a biphasic effect (Ibu-VO and Nap-VO in UMR106), or the inhibition of cell growth in a dose–response manner (Nap-VO in MC3T3E1 cells and UMR106 cells in high doses) (Figure 2). From all tested compounds, Nap-VO was the most potent inhibitor of cell growth, mainly in osteosarcoma cells (Figure 2A), as subsequently confirmed by the same research group [52]. By contrast, ibuprofen and naproxen alone, tested in the same concentration range of 0–100 µM, neither inhibited nor promoted cell proliferation.

In the case of the vanadyl(IV)–aspirin complex (Asp-VO), the non-transformed cell line was found to be more sensitive to such derivatives when compared with the osteosarcoma cell line [53]. Nevertheless, a follow-up study showed that Asp-VO was able to inhibit cell adhesion, spreading, and migration in UMR106 cells, in a mechanism dependent on protein kinase A (PKA) activity [54]. Taken together, these results highlight the need for investigating the anticarcinogenic potential of NSAIDs–VO^2+^ complexes in other types of tumors.

### 4.2. Vanadium Compounds Bound to Bisphosphonates

Bisphosphonates (BPs) are used to treat bone resorption. Both alendronate (Ale) and zoledronate (Zol) are classed as nitrogen-containing BPs. BPs can induce apoptosis, due to the production of cytotoxic ATP analogs [55,56]. In addition, BPs can inhibit cell adhesion, invasion, and proliferation; modulate the immune system, and affect angiogenesis [57]. Because of its high affinity for bone, Zol is used in the treatment of metastatic prostate bone metastases [58,59,60]. BPs also reduce bone metastasis and mortality in patients with early-stage breast cancer [61,62]. Moreover, recent evidence suggested an association between the use of BPs and reduced risk of endometrial cancer, mainly in postmenopausal women [63]. However, the known adverse effects of BPs [64,65] justify developing safe and effective bisphosphonate conjugates for adjuvant treatment of metastatic bone cancers. Indeed, many BP-conjugates containing anticancer drugs were previously tested [66], while other authors have proposed encapsulation in liposomal nanoparticles [67] to improve uptake and efficiency, and to decrease toxicity.

Hybrid vanadium-bisphosphonates (V-BPs) (Figure 1C) showed anticancer activity [43]. BPs complexed with polyoxidovanadates with nuclearities ranging from 3 to 6, V_6_(Ale)_4_, V_5_(Ale)_2_, V_5_(Zol)_2_, and V_3_(Zol)_3_, inhibited the proliferation of different tumor cell lines, such as MCF-7 (breast cancer), NCI-H460 (lung cancer), and SF-268 (glioblastoma) (Table 1) [43]. While the calculated IC_50_ values were comparable with those obtained when treating cells with decavanadate (Na_6_[V^V^_10_O_28_]), they were much lower than for the ligands themselves, especially for free alendronate (Ale), which was also considerably less potent than zoledronate (Zol) (Table 1). Nevertheless, the differences between the four V-BPs were minimal, suggesting that the BPs do not play a major role in inhibiting cell viability and that most of the activity comes from the inorganic part. Compared with other hybrid BPs, polyoxidometalates (POMs) such as with Mo^VI^ and W^VI^, the complexes containing V^IV,V^ cores, showed the greatest inhibitory potential [43].

Interestingly, both V_5_(Ale)_2_ and V_3_(Zol)_3_ complexes also showed antiparasitic potential, reducing the viability of *Leishmania tarentolae* cultures, while the ligands alone (i.e., alendronate (Ale) or zoledronate (Zol)) did not show activity against these parasites [68], highlighting the therapeutic potential of such vanadium-based compounds.

### 4.3. Metformin-Decavanadate

Metformin belongs to the biguanide group of antidiabetic drugs that have been widely used for many years [69,70]. Based on its safety profile and the current knowledge of its mechanisms of action, metformin has additional approved medical off-label indications (namely obesity and polycystic ovary syndrome) and has accumulated evidence to be repositioned for the treatment of age-related diseases (such as sarcopenia), inflammatory diseases, and cancer [71,72,73]. Almost two decades ago, the first epidemiological evidence revealed that diabetic patients taking metformin were less prone to developing cancer [74]. Metformin is, by far, the most frequently studied antidiabetic agent in clinical trials (typically combined with chemotherapy) [75]. However, it is currently debatable whether metformin as a cancer therapeutic is truly effective [73,76,77], despite new evidence regarding its potential benefits when combined with immunotherapy [78,79].

After its synthesis and characterization [80], metformin-decavanadate (Metf-V_10_) (Figure 1B) was proposed for the treatment of diabetes *mellitus*, and found to have hypoglycemic properties and an excellent safety profile in animal models [42,81,82]. Recently, it was further tested for its potential anticancer action in hepatoma and melanoma cell lines [83,84]. When compared to the decavanadate sodium salt (V_10_), a higher concentration was needed to induce 50% inhibition of Ca^2+^-ATPase enzyme activity (IC_50_) (around six-fold), although similar IC_50_ values were obtained in UACC-62 melanoma cells viability (1.3-fold higher in V_10_ (Figure 1A) compared to Metf-V_10_) [84]. In the hepatoma HepG2 cells, by contrast, a 3-fold higher IC_50_ was observed for Metf-V_10_ compared to V_10_ [83]. Despite these inconsistencies, both studies showed PI3K/AKT signaling pathways were activated by both Metf-V_10_ and V_10_ in a dose-dependent manner [83,84], suggesting that AKT hyperactivation could be one of the mechanisms of action involved, independent of the cancer cellular context.

### 4.4. Cetirizine-Based Oxidovanadium (IV) Complex

Cetirizine (CTZ) is an antihistamine medicine commonly used for treating allergic diseases. Other antihistaminic drugs showed antitumoral potential, particularly in colorectal cancer, associated with enhanced immune response [85]. Improved cancer survival was associated with the administration of the antihistamine desloratadine, specifically in patients with tumors that respond to therapy with immune checkpoint inhibitors, while lower evidence was found for CTZ, which was only observed in gastric, pancreatic, and ovarian cancer [86]. However, others showed that the concomitant use of CTZ and anti-PD-1 monoclonal antibodies led to increased progression-free survival in patients with stage IIIb-IV melanoma, suggesting that the effect of CTZ may synergize with immunotherapies enhancing its efficacy [87].

**Figure 3:**
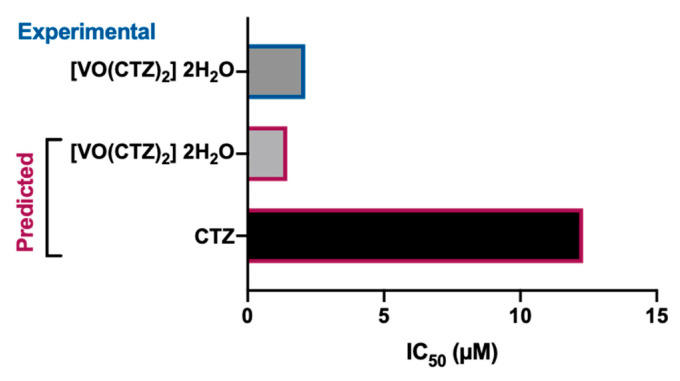
Predicted and experimental anticancer activity of cetirizine (CTZ) and [VO(CTZ)_2_] 2H_2_O in colon cancer. The IC_50_ values were extracted from [44].

Recently, the propensity for DNA binding and biological potency of different VO^2+^ complexes was evaluated by absorption titration and electrophilicity, respectively. Their behavior on a specific protein in colon cancer cells was also studied using molecular docking [44]. The cetirizine-based oxidovanadium(IV) complex ([VO(CTZ)_2_].2H_2_O) (Figure 1D) showed enhanced binding affinity to the studied protein when compared with the free ligand (i.e., CTZ). Based on the quantitative structure–activity relationships (QSAR) model, a prediction of effective activity against colon cancer was obtained for the CTZ complex (PRED IC_50_ = 1.45 μM) (Figure 3). When performing cellular in vitro experiments of cytotoxicity (sulforhodamine B method), the IC_50_ of [VO(CTZ)_2_].2H_2_O was comparable to the predicted value for the human colon cancer cell line HCT116 (2.11 μM) (Figure 3), and over 300 times higher for the normal cell line LLC-MK2 (649.8 μM). Interestingly, when compared to cisplatin (2.13 μM), the [VO(CTZ)_2_].2H_2_O complex showed similar IC_50_ values, other than presenting the highest K_b_ value (1.40 × 10^6^ M^−1^) upon DNA interaction, which implies that the compound has a better binding ability compared with other vanadium compounds and its ligands [44].

The authors of the above study also synthesized and characterized other drug-based oxidovanadium(IV) complexes, namely with carbimazole ([VO(SO_4_)(CBZ)] 8H_2_O), lornoxicam ([VO(LOR)_2_] SO_4_) and sulfonamide ([VO(SO_4_)(SCZ)] 7H_2_O), though those were considered with lower biological potency and less capacity as anticancer agents, compared to the cetirizine complex [44]. 

### 4.5. Clotrimazole (CTNZ), Miconazole (MNZ), and Pantoprazole (PNZ) Vanadyl-Based Complexes

Imidazole derivates are used as anticancer agents, namely dacarbazine and temozolomide, or zoledronic acid (*referred to in Section 4.2*), among many other drugs [88,89]. Additional examples of medicines comprising this five-member ring molecule containing a nitrogen atom include clotrimazole (CTNZ), miconazole (MNZ), and pantoprazole (PNZ), which are traditional antifungal (CTNZ, MNZ) and proton pump inhibitor (PNZ) medications. Nevertheless, there is experimental evidence they may be repositioned to treat cancers, such as hepatocellular carcinoma [90], bladder cancer [91], breast cancer [92], glioblastoma [93], gastric cancer [94], and others.

**Figure 4:**
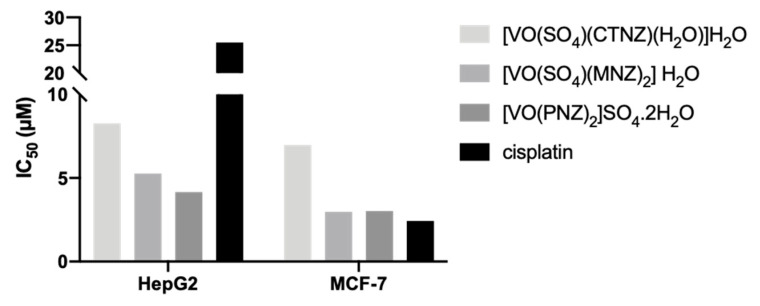
Experimental anticancer activity of the imidazole-based oxidovanadium(IV) complexes [VO(SO_4_)(CTNZ)(H_2_O)]H_2_O, [VO(SO_4_)(MNZ)_2_] H_2_O, [VO(PNZ)_2_]SO_4_.2H_2_O in HepG2 and MCF-7 cell lines. The IC_50_ values were obtained from [95]. Abbreviations: CTNZ, clotrimazole; MNZ, miconazole; PNZ, pantoprazole.

The aforementioned imidazole molecules were reacted with oxidovanadium(IV) salt and the following complexes were obtained: [VO(SO_4_)(CTNZ)(H_2_O)]H_2_O (Figure 1E), [VO(SO_4_)(MNZ)_2_] H_2_O (Figure 1F), [VO(PNZ)_2_]SO_4_.2H_2_O (Figure 1G) [95]. After treating the hepatocellular carcinoma HepG2 and the breast adenocarcinoma MCF-7 human cell lines for 24 h, all oxidovanadium(IV)-based imidazole drug complexes showed either comparable (MCF-7 cells) or lower (HepG2 cells) IC_50_ values compared to cisplatin (Figure 4), evaluated by the MTT metabolic assay [95]. When analyzing their binding affinities as targeted drug molecules with specific hepatocellular carcinoma and breast cancer proteins, the authors of the latter study obtained higher molecular docking scores for all three complexes compared to those for the free imidazole ligands [95].

## 5. Discussion

In recent years, numerous studies have been conducted with vanadium compounds, including polyoxidovanadates (POVs) and vanadium complexes incorporated into different ligands of organic origin. These compounds have been of interest in various fields, including chemistry, biology, and materials science. Due to their diverse biological activities, they have demonstrated a variety of potential applications in medicine, namely as antidiabetic, anticancer, antibacterial, and antiviral preparations [40,96,97,98]. Polyoxidometalates (POMs) have been referred to as promising anticancer agents. Decavanadate (V_10_), perhaps the most studied POM in biology, and other POMs are at the forefront as they have shown anticancer properties against several types of cancer [39,99]. However, the application of metals in biomedicine has evolved rapidly. Metal complexes and polyoxidometalates are being delivered in nanoparticles or nanocomposites containing metal compounds (metal–organic frameworks, MOFs), as these may reduce toxic effects and enhance anticancer properties. Polyoxidometalate-based metal–organic frameworks (POM-based MOFs) have also been described as promising agents against cancer [100,101,102,103]. However, the knowledge regarding the specific mechanisms of action, potential side effects, and long-term safety of vanadium in various forms, such as organic complexes, POMs, nanoparticles, or MOFs, is currently limited and requires further exploration through fundamental research and clinical studies.

The discovery and success of clinical treatment with cisplatin was a pivotal moment that sparked interest in metal complexes—that is, complexes containing metal ions (Ru, Cu, Au, and Co)—to be used as anticancer agents. Metallic complexes containing Ru, Cu, Au, Co, and V, as well as metalloids and lanthanides, were previously studied and showed anticancer potential, especially when complexed with NSAIDs [50]. Amid numerous review articles on the therapeutic applications of metal complexes, we specifically examined the potential of vanadium complexes with non-cancer drugs, known as “hard repurposing”, for cancer treatment. These are quite distinct from existing clinically approved metal-based drugs, such as cisplatin and carboplatin as anticancer chemotherapy drugs, or auranofin (containing gold(I)) for rheumatoid arthritis [104]. It is interesting, however, that auranofin also showed potential for repurposing, namely for cancer therapy or COVID-19 treatment [105,106]. Within the scope of the use of vanadium complexed with market-approved drugs, complexes with antidiabetic drugs such as metformin, hybrid vanadium with bisphosphonates used to treat bone resorption, hybrid vanadium with antifungal (CTNZ, MNZ) and proton pump inhibitors (PNZ), and complexes with NSAIDs (naproxen, aspirin), all showed anticancer results by using in silico and in vitro methodologies.

**Figure 5:**
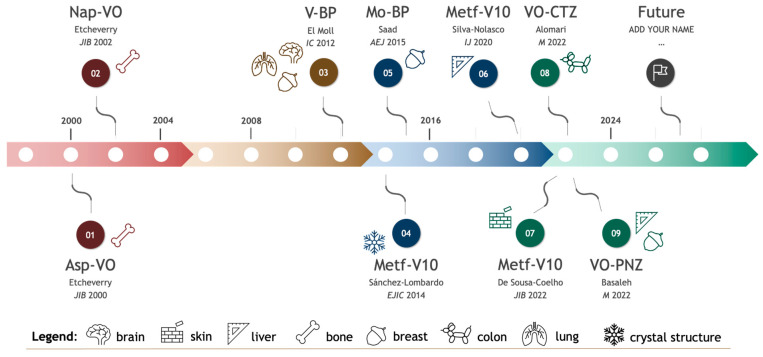
Timeline of selected complexes of marketed-approved drugs with transition metals, synthesized and characterized over the past 25 years. Chronological representation of each significant publication. For each complex represented, the last name of the first author and year of publication is shown [43,44,51,53,80,83,84,95,110]. Abbreviations: Asp-VO, vanadyl(IV)–aspirin complex; Nap-VO, vanadyl(IV) complex with naproxen; V-BP, hybrid vanadium-bisphosphonates; Metf-V10, metformin-decavanadate; Mo-BP, polyoxidomolybdate-bisphosphonates; VO-CTZ, cetirizine-based oxidovanadium(IV) complex; VO-PNZ, oxidovanadium(IV)-based pantoprazole complex.

Vanadyl(IV) complexes with NSAIDs were synthesized in the early 2000s (Figure 5), being, to the best of our knowledge, the first vanadium complexes with market-approved drugs. The effect on cellular proliferation of Asp-VO, Ibu-VO, and Nap-VO was evaluated in osteosarcoma cells [51,53], but only Nap-VO was a more potent inhibitor of the growth of cancer cells than normal osteoblast-like cells. It was only a decade later (Figure 5), that another research group evaluated the bioactivity of V-BPs, also in osteosarcoma [43]. Because of the role of vanadium in regulating phosphate metabolism, it can potentially counteract bone-related diseases [107,108], which may increase its relevance to this type of tumor. Indeed, oxidovanadium(IV) complexes also showed antitumoral properties in osteosarcoma cells, by interacting with DNA and cellular organelles such as lysosomes and mitochondria [109]. Interestingly, other polyoxometalates containing BPs were later synthesized and characterized, specifically polyoxidomolybdate-bisphosphonates (Mo_4_Zol_2_Mn) (Figure 5), which inhibited the growth of breast cancer cells [110], and bisphosphonate platinum complexes (Pt(isopropylamine)_2_(BP)]NO_3_), which in this case were tested in human prostate, ovarian, and breast cancer cell lines, but did not show relevant cytotoxic activity [111]. All the parent drugs reviewed here (*in* Section 4) have repurposing potential for cancer treatment. However, the specific types of cancer tested may not always align with the potential applications of these drug combinations.

Although synthesized and tested for its antidiabetic potential much earlier [42,80], it was not until the 2020s that the anticancer potential of metformin-decavanadate was explored, both in human hepatoma and melanoma cell lines (Figure 5) [83,84]. Recently, metformin complexed with Cu(II) ions was also evaluated for its anticancer activity [112]. It is not clear why these 10-year gaps occurred in this research topic, although there is evidence that other complexes of marketed-approved drugs with transition metals other than vanadium were synthesized [113]. Non-exhaustively, these may include complexes of pyrazinamide (PZA) (used for the treatment of tuberculosis) with Cu(II) [114] or complexes of Tolbutamide (TBA) (a sulfonylurea antidiabetic drug) with molybdenum [115], highlighting the interest in the potential application of metal complexes of pharmaceutical substances in the medical field. This is the case for the oxidovanadium(IV) complexes with cetirizine and imidazole derivates, recently developed by the Hussien research group (Figure 5), and tested against colon, breast, and hepatic cancer cells [44,95]. In addition to testing the in vitro cytotoxicity of the compounds, the authors of these two studies carried out theoretical calculations and molecular docking studies [44,95]. This use of computational approaches is of great importance in drug discovery, particularly for understanding the molecular interaction of the drugs.

There are additional vanadium compounds made with molecules with therapeutic properties. However, it is important to note that these molecules are not approved as drugs by regulatory authorities. These include oxidovanadium(IV) complexes of flavonoids, such as chrysin (Figure 1I), morin, silibinin, and quercetin, which showed antitumor properties for breast cancer and osteosarcoma, among others [40,46,116]. Other vanadium complexes with anticancer properties include phenanthroline ligands, which show antiparasitic activity [117], as well as salicylaldimines and aromatic heterocycles [118]. However, the mode of action of these vanadium complexes, as well as other metal compounds, needs to be further explored. 

Some perspectives for future studies in this area may include the optimization of vanadium complexes, especially involving the modification of the structure of vanadium complexes to increase their specificity and anticancer activity; the use of combination therapies that pair vanadium-based compounds with existing or repurposed anticancer drugs to improve their effectiveness; and ideally, by a patient-specific approach exploring the use of vanadium complexes in personalized treatment plans based on the patient’s unique genetic and molecular profiles.

## 6. Conclusions

To date, no drug complexed with vanadium has received regulatory approval for clinical use in the treatment of cancers. Indeed, available studies involving vanadium–approved drug association are still relatively scarce. However, research into vanadium and POVs-based compounds for cancer therapy is ongoing and several compounds and approaches are being explored, namely vanadium-based complexes with repurposed common drugs such as metformin, cetirizine, or naproxen. This is a strategy that should be developed in the coming years, as this association may improve both the anticancer activity of vanadium and the therapeutic efficacy of the current approved/repurposed drugs.

## Figures and Tables

**Table 1:** Human tumor cell growth inhibition upon vanadium complexes with approved drugs and for decavanadate. IC_50_ (µM) determined by MTT ((3-(4,5-dimethylthiazole-2-yl)-2,5-diphenylte-trazolium bromide) cell proliferation assay. The values were collected from [43].

Treatment	NCI-H460 Cells	MCF-7 Cells	SF-268 Cells
V_6_(Ale)_4_	0.4 ± 0.0	0.5 ± 0.3	0.5 ± 0.2
V_5_(Ale)_2_	0.5 ± 0.1	0.5 ± 0.2	0.8 ± 0.2
Ale	200 ± 43	130 ± 2.2	140 ± 13
V_5_(Zol)_2_	0.5 ± 0.1	0.4 ± 0.2	0.4 ± 0.0
V_3_(Zol)_3_	0.3 ± 0.2	0.3 ± 0.0	0.3 ± 0.2
Zol	8.1 ± 1.7	7.7 ± 2.6	12.4 ± 1.4
Na_6_[V_10_O_28_]	0.2 ± 0.1	0.3 ± 0.2	0.3 ± 0.1

## Data Availability

Data sharing is not applicable.
